# Gender and Height Are Associated With Life Satisfaction Through Psychosocial Factors: Findings From Sweden

**DOI:** 10.1111/sjop.70000

**Published:** 2025-07-09

**Authors:** Filip Fors Connolly

**Affiliations:** ^1^ Department of Sociology Umeå University Umeå Sweden

**Keywords:** gender differences, height, life satisfaction, psychosocial factors

## Abstract

This study examines the interplay between gender, height, and life satisfaction in the Swedish population. While height consistently shows a positive correlation with life satisfaction, gender effects on life satisfaction are typically null or inconsistent across studies, suggesting complex underlying mechanisms. Using data drawn from a representative cross‐sectional sample (*n* = 990), we applied structural equation modeling (SEM) and multi‐group confirmatory factor analysis. We investigated how five psychosocial factors assessed via multi‐item scales (perceived safety, social trust, social support, social status, and financial satisfaction) mediate the effects of height and gender on life satisfaction. SEM results indicated that height and gender indirectly influence life satisfaction via the psychosocial factors, with no significant direct effects observed. Height demonstrated significant positive indirect effects on life satisfaction through perceived safety and financial satisfaction. Being female was associated with positive indirect effects on life satisfaction via social trust and social support (independent of height) but also showed negative indirect effects through height‐mediated pathways involving safety and financial satisfaction. The findings suggest that height's positive association with life satisfaction operates indirectly via psychosocial factors, particularly perceived safety and financial satisfaction. Simultaneously, the lack of a direct gender‐life satisfaction relationship may stem from counterbalancing indirect pathways, with some male advantages potentially operating through height. This study highlights the complex interplay of physical characteristics and psychosocial experiences in shaping well‐being.

**Summary**

- The relationships between height, gender, and life satisfaction were entirely indirect, mediated by psychosocial factors.
- Height's positive association with life satisfaction was mediated by higher perceived safety and greater financial satisfaction.
- The absent direct link between gender and life satisfaction was explained by counterbalancing indirect effects.
- Positive pathways for women (via social support and trust) were offset by negative, height‐mediated pathways related to safety and financial satisfaction.

## 1 Introduction

The study of subjective well‐being in terms of life satisfaction has become increasingly central to social scientists, offering crucial insights into societal progress and individual quality of life (Helliwell et al. 2017). Scholars have identified a wide array of factors contributing to life satisfaction, ranging from macro‐level societal factors to individual socioeconomic conditions, personal characteristics, and psychosocial factors (Diener et al. 2018).

Previous research has revealed a complex interplay between psychosocial factors, individual characteristics, and life satisfaction. For instance, Fors Connolly and Johansson Sevä (2021) demonstrated that psychosocial factors, specifically perceptions of social status and social inclusion, help explain why extraversion consistently predicts life satisfaction. However, while psychological studies have explored mechanisms between personality traits and life satisfaction, less attention has been given to how more objective individual characteristics relate to life satisfaction through psychosocial factors.

In this article, we examine how gender and height relate to life satisfaction in a Swedish context, focusing on five key psychosocial factors as possible mediators. Our choice to study gender and height is important for several reasons. First, both are stable individual traits that remain largely unchanged throughout adulthood, eliminating concerns about reverse causality. Second, they are substantially correlated, necessitating a simultaneous examination to disentangle their unique effects. Third, both are readily observable and play significant roles in social interactions, influencing how individuals are perceived and treated by others, thus highlighting their potential to influence psychosocial experiences. Lastly, while both have been studied in relation to life satisfaction, few studies have attempted to analyze the mechanisms underlying these relationships and the interplay of gender and height on life satisfaction.

Interestingly, while both gender and height have a clear relationship with socioeconomic outcomes, their relationship with life satisfaction has been more mixed. Several studies have documented a positive relationship between height and life satisfaction, often attributing this to socioeconomic benefits (Deaton and Arora 2009; Habibov et al. 2020; Carrieri and De Paola 2012; Sohn 2016). In contrast, despite extensive research on gender inequalities across various life domains, studies have generally found no consistent direct relationship between gender and life satisfaction in most countries (Batz‐Barbarich et al. 2018; Joshanloo and Jovanović 2020). This apparent contradiction raises important questions about how individual characteristics influence life satisfaction. We propose that a potential explanation lies in the complex interplay of psychosocial factors, which may mediate the relationships between height, gender, and life satisfaction. Specifically, we focus on five key psychosocial factors that can be linked to height, gender, and life satisfaction: perceptions of social trust, social support, social status, financial satisfaction, and safety. Our selection of these five specific psychosocial factors is theoretically driven, as they represent distinct domains of life potentially influenced by both height and gender, covering relational, economic, hierarchical, and physical security dimensions of human experience.

We hypothesize that height positively affects all five psychosocial factors, while gender displays mixed relations. We further hypothesize that height may mediate some gender differences in psychosocial factors. In the following sections, we first present a theoretical framework detailing the relationships between individual characteristics, psychosocial factors, and life satisfaction. We then describe our methodology, present our findings, and discuss their implications for understanding the complex pathways through which gender and height relate to life satisfaction.

## 2 Psychosocial Factors as Mediators Between Individual Characteristics and Life Satisfaction

Psychosocial factors, encompassing the interplay between psychological processes and social experiences, are crucial for understanding how individuals perceive and interact with their social environment (Martikainen et al. 2002). We define psychosocial factors as subjective perceptions and experiences of one's social and personal environment, distinct from objective life circumstances. Extensive research demonstrates that these factors mediate the relationships between individual characteristics, life circumstances, and outcomes such as health, job satisfaction, and life satisfaction. For example, perceived control and social support mediate the link between socioeconomic status and health outcomes (Marmot et al. 1991; Taylor and Seeman 1999), while job characteristics like demand, control, and social support influence job satisfaction and well‐being (Theorell 1992).

**FIGURE 1:**
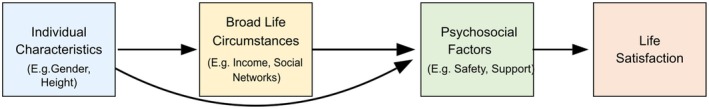
Conceptual framework: individual attributes, psychosocial factors, and life satisfaction.

Despite this extensive research, few studies have examined how specific individual characteristics, such as gender and height, relate to psychosocial factors and in turn influence life satisfaction. This gap is particularly noteworthy given that height consistently emerges as a stronger predictor of life satisfaction than gender in previous studies. Thus, understanding the mechanisms underlying these relationships is essential for a more complete understanding of life satisfaction determinants. Our proposed causal chain begins with individual characteristics influencing life circumstances, which then affect psychosocial factors, ultimately impacting life satisfaction (see Figure 1). We conceptualize life circumstances as objective conditions such as socioeconomic factors (e.g., income, education, occupational status) and structural social opportunities (e.g., social networks). These objective conditions then shape psychosocial factors, such as perceived social support and social status recognition, that serve as proximal determinants of life satisfaction. Additionally, individual characteristics may directly affect psychosocial factors independent of life circumstances. For instance, physical attributes associated with gender and height, such as muscle mass and strength, can influence feelings of physical vulnerability and perceived safety (Manson et al. 2023). Height and gender may also directly influence perceptions of social status and social support. For example, taller individuals and men may be perceived as more dominant (Judge and Cable 2004), enhancing their social status and influencing social interactions regardless of socioeconomic factors. Internalized gender roles (Eagly et al. 2000) and intrinsic personality differences (Falk and Hermle 2018) may shape social behaviors and emotional expressiveness, affecting the ability to form and maintain supportive relationships independently of living conditions.

### 2.1 Height, Psychosocial Factors and Life Satisfaction

In this study, we hypothesize that height is positively associated with all five psychosocial factors, leading to higher life satisfaction. Although previous research has linked height to favorable life circumstances such as high income and occupational standing (Judge and Cable 2004; Case and Paxson 2008; Thompson et al. 2023) less research has comprehensively analyzed how height relates simultaneously to a broader set of psychosocial factors, including social trust, social support, social status, financial satisfaction, and perceived safety, particularly as mediators of the relationship between height and life satisfaction. Building on existing findings, we examine these potential pathways through a systematic analysis of each factor.

For social status, the evidence is particularly robust. Height is consistently positively associated with occupational prestige and income (Judge and Cable 2004; Case and Paxson 2008; Thompson et al. 2023), which may directly contribute to perceptions of social status. Moreover, taller individuals are often perceived as more dominant and leader‐like (Blaker et al. 2013), enhancing their sociometric status, the respect and admiration received from others, independent of socioeconomic achievements. Moreover, Judge and Cable (2004) found the correlation between height and subjective perceptions of social esteem was particularly robust, exceeding correlations with more objective metrics such as performance or leader emergence. As perceived social status predicts life satisfaction (Fors Connolly and Johansson Sevä 2018), this represents a first pathway linking height to life satisfaction.

Given this robust evidence linking height to social status, and the established role of status in facilitating supportive social relationships, it is plausible that height also influences social support. Taller individuals may thus form stronger and more supportive social connections. Although direct empirical evidence for this association is limited, it is theoretically grounded in the enhanced perceived status and competence associated with height (Stulp et al. 2013), which may promote social network formation and maintenance. Considering that perceived social support consistently predicts life satisfaction across cultures (Siedlecki et al. 2014), this represents a second potential pathway linking height to life satisfaction.

For financial satisfaction, the mechanism appears straightforward. The well‐documented “height premium” in labor markets translates to higher earnings and better occupational positions for taller individuals (Judge and Cable 2004; Thompson et al. 2023). These objective economic advantages likely contribute to greater subjective financial satisfaction, which is associated with overall life satisfaction (Habibov et al. 2020). Furthermore, these economic advantages associated with height appear persistent throughout individuals' careers, suggesting a stable, long‐term pathway influencing financial satisfaction and potentially overall well‐being (Judge and Cable 2004). This economic pathway represents a third route through which height may enhance well‐being.

Regarding social trust, experimental research using virtual reality has demonstrated that reduced height manipulations increase paranoia, negative social comparisons, and perceived vulnerability (Freeman et al. 2014). Thus, greater height likely fosters higher social trust by reducing feelings of vulnerability and promoting positive self‐perception during social interactions. Similarly, we propose that taller individuals may also experience greater perceived safety due to reduced physical vulnerability. Height is plausibly seen, both by oneself and others, as conferring defensive advantages or deterring potential aggression, enhancing an individual's overall sense of security. As both social trust (Helliwell et al. 2016) and perceived safety (Tay and Diener 2011) are positively associated with life satisfaction, these mechanisms represent additional pathways, fourth and fifth, respectively, linking height to life satisfaction.

### 2.2 Gender, Psychosocial Factors and Life Satisfaction

While we hypothesize consistently positive associations between height and all five psychosocial factors contributing to higher life satisfaction, gender appears to present a more complex relationship involving both advantages and disadvantages across these domains, potentially explaining the lack of consistent gender differences in overall life satisfaction found in previous research.

For social support, the evidence indicates an advantage for women. Research demonstrates higher satisfaction and intimacy in women's friendships (Jones 1991; Elkins and Peterson 1993), with women reporting more emotionally supportive relationships (Aukett et al. 1988) and less loneliness than men (Barreto et al. 2021). This gender difference aligns with Social Role Theory, where women's greater emphasis on nurturing relationships and emotional sharing stems from gender socialization processes (Cross and Madson 1997; Eagly et al. 2000). Empirical studies consistently show that women report larger support networks and higher perceived social support (Coventry et al. 2004; Milner et al. 2016), a pattern confirmed by a recent meta‐analysis showing that females both give and receive significantly more social support across online environments (Tifferet 2020). Given the robust evidence and theoretical grounding, we hypothesize that women report higher social support than men, providing women with an advantage in this psychosocial domain. As higher social support consistently predicts higher life satisfaction (Siedlecki et al. 2014), this hypothesized advantage represents a potential pathway through which being female may positively influence life satisfaction.

Regarding social trust, the evidence presents a complex picture. Theoretical perspectives, including Social Role Theory and Parental Investment Theory, suggest women may exhibit lower trust due to higher sensitivity to risk and betrayal, while men may demonstrate higher trust as a strategy for resource acquisition (Van den Akker et al. 2020). Empirical evidence, however, is ambiguous: some cross‐national studies find men to be more trusting than women (Norris and Inglehart 2006), while some studies suggest the opposite (Falk and Hermle 2018; Hooghe et al. 2009), and yet others report no significant gender differences in generalized trust (Kaasa and Parts 2008). Mewes (2014) explains the mixed findings by demonstrating that gender differences in trust depend on contextual factors such as gender equality in labor force participation. Specifically, gender differences in trust become negligible in countries with high employment equality, such as Sweden, but persist (lower trust among women) in countries characterized by larger gender gaps in labor force participation. Given Sweden's highly equal labor force participation, these findings support our hypothesis that there are no significant overall gender differences in social trust. Although social trust strongly predicts life satisfaction (Helliwell et al. 2016), the lack of a hypothesized direct gender difference in trust leads us to hypothesize no significant indirect effect of gender on life satisfaction via this pathway in the Swedish context.

Concerning financial satisfaction, we anticipate disadvantages for women. Despite progress in recent decades, women continue to face significant barriers in income and wealth accumulation (Blau and Kahn 2017), potentially lowering their financial satisfaction. Empirical literature largely supports this expectation. Several studies, including analyses using U.S. and Croatian populations, report that men tend to have higher financial satisfaction than women. For instance, Fan and Babiarz (2019) found that U.S. women reported lower financial satisfaction than men, a gap that persisted across different marital statuses. Similarly, Škreblin Kirbiš et al. (2017) found Croatian men reported significantly higher financial satisfaction than women. While the cultural and institutional context of Sweden, with its relatively strong welfare system and high gender equality, may reduce gender disparities compared to other nations, persistent economic inequalities still exist (Zahidi 2024). Therefore, even within the Swedish context, we hypothesize that men report higher overall financial satisfaction than women. Given that financial satisfaction is a strong predictor of life satisfaction (Habibov et al. 2020), this hypothesized disadvantage represents a potential pathway through which being female may negatively influence life satisfaction.

For social status, both theoretical and empirical evidence suggests disadvantages for women. Women on average rate their perceived status lower than men (Fors Connolly and Johansson Sevä 2018), likely reflecting both objective socioeconomic disadvantages and subjective experiences of diminished respect and admiration in social groups. Social Role Theory positions men as typically higher in agency, traits associated with status and power, and women higher in communion (Eagly et al. 2000). Additionally, a large body of research documents a “male hubris, female humility” effect, i.e., men systematically overestimate, and women underestimate, their own abilities (e.g., Furnham et al. 2001; Szymanowicz and Furnham 2011). This tendency likely amplifies gender differences in perceived social status, contributing to an inflated sense of male superiority. Thus, we hypothesize that men report higher perceived social status than women, representing another psychosocial disadvantage for women. As higher perceived social status also predicts higher life satisfaction (Fors Connolly and Johansson Sevä 2018), this represents another potential pathway through which being female may negatively influence life satisfaction.

Regarding perceived safety, research consistently shows women report lower safety perceptions than men, particularly in public spaces (O'Brien 2005; Jorgensen et al. 2013; Steinmetz and Austin 2014; Madge 1997), a phenomenon often termed the “gender‐fear paradox” as it persists despite women's lower victimization rates for many crime types (Hummelsheim‐Doss and Lysova 2023). Women's greater perceived physical vulnerability and heightened sensitivity to environmental cues of danger contribute to this disparity. Recent research demonstrates that physical strength mediates sex differences in fearful and anxious personality traits, with women's lower average strength contributing to higher levels of fear and anxiety compared to men (Manson et al. 2023). Therefore, we hypothesize that men report higher perceived safety than women. Since perceived safety is strongly linked to higher life satisfaction, this expected gender difference constitutes a further potential pathway through which being female may negatively influence life satisfaction.

Despite the hypothesized psychosocial disadvantages for women in financial satisfaction, social status, and perceived safety, the advantages in social support and the potentially neutral effects of gender on social trust may help explain why gender often shows no consistent direct relationship with overall life satisfaction. Additionally, social comparison processes may play a role, with women possibly comparing themselves primarily to other women rather than men (Batz and Tay 2018), potentially buffering the impact of psychosocial disadvantages on life satisfaction.

An additional aspect of our framework is the potential role of height in mediating gender differences across multiple psychosocial factors. Given that research indicates taller stature confers labor market advantages, with studies suggesting that the positive effect of height on earnings and overall workplace success applies significantly to both men and women (Judge and Cable 2004; see also Thompson et al. 2023), we propose that the average difference in height between men and women may partially mediate observed gender disparities in related psychosocial factors like financial satisfaction and social status. While the impact per unit of height on certain success metrics may be roughly comparable across genders, the baseline difference in average height could still contribute to differing average psychosocial experiences linked to status and economic standing. Thus, we hypothesize a negative indirect effect of being female on life satisfaction operating via height and financial satisfaction, and similarly via height and social status. Since height correlates with physical strength, which in turn influences perceived vulnerability (Manson et al. 2023), we hypothesize that average height differences partially mediate gender disparities in perceived safety. This translates to a hypothesized negative indirect effect of being female on life satisfaction operating via height and perceived safety. This height‐mediation hypothesis offers a new perspective on how a physical characteristic may contribute to gender differences in psychosocial experiences and, ultimately, life satisfaction.

Our hypotheses are summarized in Table 1. We aim to provide a comprehensive analysis of the relationships among gender, height, and five key psychosocial factors, offering new insights into the psychosocial advantages and disadvantages associated with gender and height that have not been previously studied. Additionally, we conduct a mediation analysis to test whether these psychosocial factors can explain why height has shown a more consistent relationship with life satisfaction than gender in previous studies. Finally, we examine whether height may mediate some of the effects of gender on psychosocial factors and life satisfaction.

**TABLE 1:** Summary of proposed mediation hypotheses.

	Height → Mediator → LS	Gender → Mediator → LS	Gender → Height → Mediator → LS
Social trust	Positive effect	No effect	No effect
Social support	Positive effect	Positive effect (female)	No effect
Social status	Positive effect	Negative effect (female)	Negative effect (female)
Financial satisfaction	Positive effect	Negative effect (female)	Negative effect (female)
Safety	Positive effect	Negative effect (female)	Negative effect (female)
Direct effects on LS	No effect	No effect	

## 3 Method

### 3.1 Participants and Procedure

To examine the relationships between gender, height, psychosocial factors, and life satisfaction, we used data from a combined online and paper‐and‐pencil survey sent to a representative sample of the Swedish population aged 15 years or older. The questionnaire included a wide range of topics, with an added set of questions specifically related to subjective well‐being. As a result, many questions unrelated to the primary research question were also included in the survey. A stratified sampling technique was employed, with random samples drawn from each of the eight NUTS‐2 regions in Sweden, ensuring a geographically diverse and representative sample.

A total of 1500 individuals were invited to participate and offered a conditional incentive of SEK 300 in the form of gift cards, while an additional 1500 participants were invited without being offered any incentive. The purpose of offering incentives to one group and not to the other was to investigate potential non‐response bias effects. Since this aspect was not directly relevant to this study, the two samples were combined for subsequent analyses to leverage the full dataset. The fieldwork was conducted between June and December of 2023. The final net sample consisted of 1074 individuals (35.8% response rate). For the analyses presented in this study, we retained only participants who provided complete responses to all relevant survey questions (*n* = 992) and excluded two cases with outlier height values (60 and 213 cm) as they represented clear breaks in the distribution. The remaining heights in our sample ranged from 146 cm to 200 cm, following an expected normal distribution for adult height, resulting in a final analytical sample of 990 participants. As detailed in Appendix Table A1, this sample comprised slightly more females (52.8%) than males (47.2%), represented a wide age distribution, with the largest concentration of participants in the 55–74 year age group (34.5%), and was predominantly composed of individuals born in Sweden (83.7%). The largest proportion of participants were married or in a civil partnership (44.1%) and fell into the middle (43.0%) or highest (40.4%) household income groups.

The initial recruitment target of 3000 invitations was set based on practical considerations, rather than an a priori power analysis to determine the minimum required sample size for the specific analyses in this paper. However, to ensure the adequacy of the achieved sample for detecting meaningful effects in our planned analyses, post hoc power calculations were performed. These calculations demonstrated sufficient statistical power: 88% power to detect correlations of *r* = 0.10 and 91% power to detect small effects (*f* ^2^ = 0.02) in regression models with 8 predictors (*α* = 0.05; Faul et al. 2007). These power levels exceed the conventional 80% threshold, indicating adequate sample size for the primary analyses conducted in this study.

### 3.2 Measures

Life satisfaction was assessed using the following two items: “Imagine a ladder with steps numbered from 0 at the bottom to 10 at the top. Assume that the top of the ladder represents the best possible life for you, and the bottom of the ladder represents the worst possible life for you. If the top step is 10 and the bottom step is 0, on which step of the ladder do you personally stand right now?” and “Please rate your level of satisfaction with your life as a whole on a scale from 0 to 10, with 0 being ‘Extremely dissatisfied’ and 10 being ‘Extremely satisfied’.” We employed two bipolar items to reduce the response burden while capturing evaluations that span from negative to positive well‐being (*α* = 0.78).

Psychosocial factors were measured with five multi‐item scales. Social trust was assessed with three items adapted from Bartolini and Sarracino (2014; *α* = 0.83) rated on a 0–10 scale (0 = “No trust at all”, 10 = “Complete trust”). Safety was captured with two items (*α* = 0.62) on a 5‐point Likert‐type scale. Social support comprised two 5‐point items (“Strongly disagree”–“Strongly agree”) plus one item asking for the number of close relations on a 7‐point scale (“None”–“10 or more”); responses were standardized before aggregation (*α* = 0.66). Financial satisfaction (three items, *α* = 0.70) and social status (two items, *α* = 0.76) used parallel 4‐ or 5‐point Likert‐type formats, with higher scores reflecting greater satisfaction or status. All items were coded so that higher values represented more of the construct. Table 2 lists full item wordings. These item sets served as indicators of latent factors in the confirmatory factor analysis reported in Section 3.

**TABLE 2:** Description of psychosocial factors used in the present study.

Factor	Key characteristics of the factor	Survey items
Financial satisfaction	Perception of financial stability and material well‐being	My economic resources are good I have a high material living standard Which of the descriptions below comes closest to how you feel about your household's income nowadays?
Safety	Sense of personal security in one's environment	How safe do you or would you feel walking alone in the area you live after dark? I feel safe
Social support	Availability and perception of support from one's social network	I have good support from relatives, friends, and coworkers People in my surroundings are there for me How many people, if any, are there with whom you can discuss intimate and personal matters?
Social status	Perceived standing or prestige relative to others	I am more successful than others I have achieved more than others in my age group
Social trust	Belief in the general trustworthiness and fairness of others	Generally speaking, would you say that most people can be trusted, or that you can't be too careful in dealing with people? Do you think that most people would try to take advantage of you if they got the chance, or would they try to be fair? Would you say that most of the time people try to be helpful or that they are mostly looking out for themselves?

Given the reliance on self‐report measures for both psychosocial factors and life satisfaction, Harman's single‐factor test was conducted as an initial check for potential common method bias (Podsakoff et al. 2003). While this test is not considered definitive due to its known limitations, it can provide an indication of whether such bias is a major concern. The first factor extracted accounted for only 30.36% of the variance, suggesting that CMB was likely not a pervasive issue in this dataset.

Demographic variables were assessed as follows: Gender was determined by asking “What gender are you?” with response options “male” or “female” (coded as 0 = male, 1 = female). Height was measured by asking “How tall are you without shoes?” with participants recording their answer in centimeters. Age and immigrant status were used as confounders in the analysis. Age was calculated based on the response to “What year were you born?” with answers recoded to reflect current age. Immigrant status was ascertained through the question “Were you born in Sweden?” with response options “Yes” or “No” (coded as 0 = born in Sweden, 1 = not born in Sweden).

### 3.3 Analysis Plan

To examine the relationships between gender, height, psychosocial factors, and life satisfaction, we conducted a series of analyses using confirmatory factor analysis (CFA) and structural equation modeling (SEM) with the Lavaan package in R (Rosseel 2012). SEM was chosen for its ability to simultaneously estimate multiple relationships while accounting for measurement error in latent constructs.

First, we constructed a measurement model using CFA. The model included six latent factors: five psychosocial constructs (social trust, social support, safety, financial satisfaction, and social status) and life satisfaction. Each latent factor was indicated by its respective observed items. We evaluated the measurement model's fit using standard fit indices: chi‐square (*χ* ^2^), comparative fit index (CFI), Tucker‐Lewis index (TLI), root mean square error of approximation (RMSEA), and standardized root mean square residual (SRMR). To assess discriminant validity between the latent constructs, we used the Fornell‐Larcker criterion (Fornell and Larcker 1981), which compares the square root of each construct's average variance extracted (AVE) with its correlations with other constructs. To assess measurement invariance across genders, we tested for configural, metric, scalar and strict invariance. This involved comparing reductions in model fit indices based on the recommendation by Chen (2007).

After establishing a satisfactory measurement model, we proceeded with the structural model. Gender and height were included as observed exogenous variables, while the psychosocial factors and life satisfaction were treated as latent endogenous variables. We specified direct paths from gender and height to life satisfaction, as well as indirect paths mediated through the psychosocial factors. Age and immigrant status were included as covariates to control for potential confounding effects. In the structural model, we estimated direct effects of gender and height on the psychosocial factors and life satisfaction, as well as the effects of psychosocial factors on life satisfaction. This allowed us to examine potential mediating pathways through which gender and height might indirectly influence life satisfaction. Given the simultaneous testing of multiple indirect pathways (fifteen specific indirect effects across the five mediators for height and gender), we applied the Benjamini‐Hochberg procedure to control the False Discovery Rate (FDR) at *q* = 0.05 for these specific tests.

## 4 Results

### 4.1 Testing a Measurement Model

The measurement model demonstrated good fit to the data, *χ* ^2^(75) = 231.954, *p* < 0.001, CFI = 0.968, TLI = 0.955, RMSEA = 0.046 (90% CI [0.039, 0.053]), SRMR = 0.036. Factor loadings were moderate to strong (see Appendix Table A2). Discriminant validity was established using the Fornell‐Larcker criterion, with the square root of AVE for each construct exceeding its correlations with all other constructs (see Appendix Table A3). Furthermore, the model met all levels of measurement invariance across gender groups, as evidenced by the small decreases in fit indices (see Appendix Table A4), indicating that men and women interpret and respond to the latent constructs similarly.

Correlations between all variables are displayed in Table 3. As expected, gender showed a strong negative correlation with height (r = −0.70, *p* < 0.001), reflecting the anticipated height difference between men and women in the sample. The correlation between height and life satisfaction was 0.11 (*p* < 0.01), while the corresponding correlation between gender and life satisfaction was −0.03 (*p* > 0.05, non‐significant). Height demonstrated modest or weak but mostly statistically significant positive correlations with the proposed mediators. The strongest associations were observed with social status (*r* = 0.22, *p* < 0.001) and safety (r = 0.21, *p* < 0.001), followed by financial satisfaction (*r* = 0.17, *p* < 0.001). Weak and non‐significant correlations were found between height and social support (*r* = 0.02, *p* > 0.05) and social trust (*r* = 0.02, *p* > 0.05).

**TABLE 3:** Correlations between gender, height, psychosocial factors, and life satisfaction.

Variable	1	2	3	4	5	6	7	8	9	10
1. Life satisfaction										
2. Social trust	0.46***									
3. Safety	0.60***	0.42***								
4. Social support	0.46***	0.34***	0.43***							
5. Financial satisfaction	0.61***	0.27***	0.46***	0.35***						
6. Social status	0.35***	−0.00	0.23***	0.19***	0.49***					
7. Gender (female)	−0.03	0.06	−0.13***	0.12***	−0.13***	−0.22***				
8. Height	0.11**	0.02	0.21***	0.02	0.17***	0.22***	−0.70***			
9. Immigrant	−0.13***	−0.11**	−0.13***	−0.18***	−0.11**	0.10**	0.04	−0.15***		
10. Age	0.19***	0.15***	0.04	−0.17***	0.10**	−0.00	−0.05	−0.02	−0.09**	

*Note:* *p < 0.05, **p < 0.01, ***p < 0.001.

Gender showed significant correlations with most of the proposed mediators, though the relationships varied in direction and strength. The strongest association was observed with social status (*r* = −0.22, *p* < 0.001), indicating that women tend to report lower status. Gender was also negatively correlated with safety (*r* = −0.13, *p* < 0.001) and financial satisfaction (*r* = −0.13, *p* < 0.001). Conversely, gender showed a positive correlation with social support (*r* = 0.12, *p* < 0.001). However, the correlation between gender and social trust was not statistically significant (*r* = 0.06, *p* > 0.05).

Life satisfaction showed stronger and more consistent correlations with the proposed mediators compared to height and gender. The strongest associations were observed with financial satisfaction (*r* = 0.61, *p* < 0.001) and safety (*r* = 0.60, *p* < 0.001), followed by social trust (*r* = 0.46, *p* < 0.001), social support (*r* = 0.46, *p* < 0.001), and social status (*r* = 0.35, *p* < 0.001). These findings indicate that individuals reporting higher life satisfaction tend to experience greater feelings of financial satisfaction, safety, social trust, social support, and perceived social status.

Correlations were also examined separately by gender (see Appendix Tables A5a and A5b). While many patterns were similar, this exploratory analysis revealed some notable differences, such as the Height‐Social Trust correlation appearing significant only for women and Height‐Financial Satisfaction/Status correlations appearing stronger for men in bivariate analyses.

### 4.2 Testing a Structural Model

**FIGURE 2:**
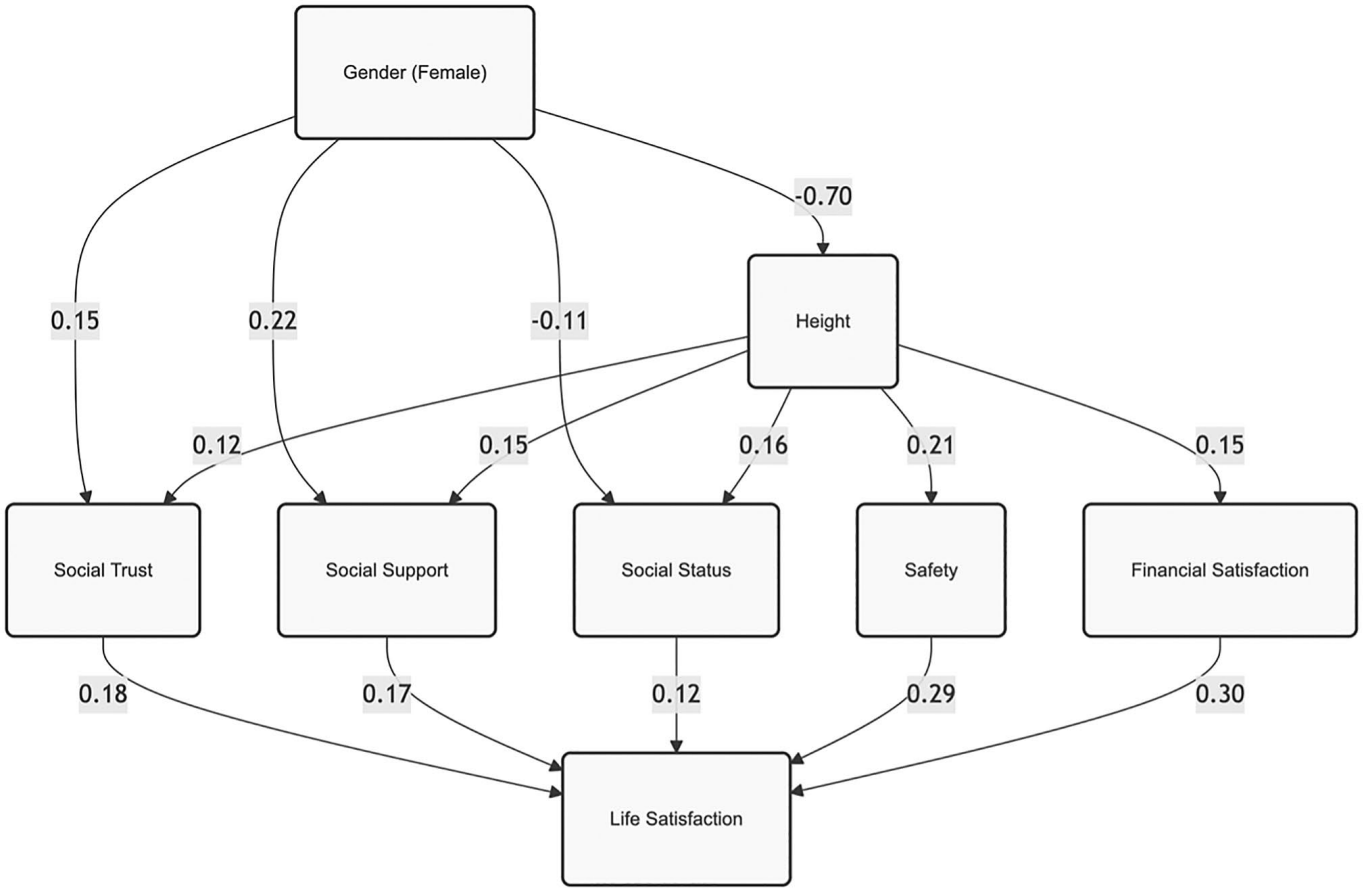
Structural equation model of gender, height, psychosocial factors, and life satisfaction. All reported coefficients are standardized beta coefficients. Only statistically significant coefficients are displayed. The model includes correlations among all latent factors; however, these correlations are not displayed in the figure to maintain visual clarity.

We tested a model examining how gender and height relate to life satisfaction, both directly and as mediated through the various psychosocial factors. This model allows us to disentangle the pathways through which these individual characteristics may influence life satisfaction and to what extent psychosocial factors explain these relationships. Importantly, this model provides multivariate associations between the variables, i.e., showing how gender and height are uniquely related to life satisfaction and the psychosocial factors, as well as the unique variance between each psychosocial factor and life satisfaction. Statistically significant standardized path coefficients are illustrated in Figure 2, while the complete set of model parameters, including non‐significant paths and the confounders, is presented in Appendix Table A6.

Our results show that gender has significant direct effects on several psychosocial factors. Being female was positively associated with social trust (*β* = 0.153, *p* = 0.001) and social support (*β* = 0.222, *p* < 0.001) but negatively associated with social status (*β* = −0.114, *p* = 0.023). Gender did not have significant direct effects on safety (*β* = 0.014, *p* = 0.770) or financial satisfaction (*β* = −0.021, *p* = 0.680). Height showed significant positive associations with all psychosocial factors: safety (*β* = 0.207, *p* < 0.001), social trust (*β* = 0.120, *p* = 0.013), social support (*β* = 0.148, *p* = 0.004), financial satisfaction (*β* = 0.145, *p* = 0.004), and social status (*β* = 0.157, *p* = 0.002). Regarding life satisfaction, neither gender (*β* = 0.065, *p* = 0.107) nor height (*β* = 0.015, *p* = 0.706) showed significant direct effects. However, all psychosocial factors were significantly associated with life satisfaction. Financial satisfaction showed the strongest relationship (*β* = 0.304, *p* < 0.001), followed by safety (*β* = 0.286, *p* < 0.001), social trust (*β* = 0.175, *p* < 0.001), social support (*β* = 0.167, *p* < 0.001), and social status (*β* = 0.118, *p* = 0.005).

In sum, the results suggest multiple pathways through which gender and height might indirectly influence life satisfaction. While gender and height don't directly predict life satisfaction, they are associated with psychosocial factors that, in turn, significantly predict life satisfaction. The positive associations of being female with social trust and social support might contribute positively to life satisfaction, while the negative association with social status might have the opposite effect. Similarly, height's positive associations with all psychosocial factors suggest potential indirect positive effects on life satisfaction. The lack of a significant direct effect of gender on life satisfaction, coupled with its varied associations with psychosocial factors, supports the idea that various positive and negative pathways may largely offset each other, resulting in a non‐significant total effect of gender on life satisfaction. These results were robust despite controlling for age and immigrant status as potential confounders.

To formally assess mediation by height and the psychosocial factors, we fitted a structural equation model with maximum‐likelihood estimation and bootstrapping (5000 resamples) to derive bias‐corrected confidence intervals and *p*‐values for each indirect effect. To address the risk of inflated type I error across the fifteen specific indirect pathway hypotheses outlined in Table 1, we applied a Benjamini–Hochberg false‐discovery‐rate procedure at *q* = 0.05. The confirmation status of hypotheses discussed below refers to significance after this FDR correction, with results summarized in Table 4.

**TABLE 4:** Summary of hypothesis testing results.

	Height → Mediator → LS	Gender (female) → Mediator → LS	Gender (female) → Height → Mediator → LS
Social trust	0.021	0.027*	−0.015
Social support	0.025	0.037*	−0.017
Social status	0.018	−0.013	−0.013
Financial satisfaction	0.044*	−0.006	−0.031*
Safety	0.059*	0.004	−0.041*
Direct effects	Height → LS: 0.015	Gender → LS: 0.065	—

*Note:* Values are standardized beta coefficients (*β*) for the specific indirect effect. Asterisk (*) indicates statistical significance after Benjamini‐Hochberg correction controlling FDR at *q* = 0.05 (based on *p*‐values from bootstrap analysis, *n* = 990). Indirect effects without an asterisk were not significant after BH correction (*p* > BH threshold). LS=Life Satisfaction.

With respect to the indirect effects of height on life satisfaction (Height → Mediator → Life Satisfaction pathway), the analysis provided partial support for our hypotheses. Consistent with predictions, significant positive indirect effects remained significant after FDR correction for the pathways via safety (*β* = 0.059, *p* = 0.003) and financial satisfaction (*β* = 0.044, *p* = 0.012). However, contrary to hypotheses, the positive indirect pathways proposed through social support (*β* = 0.025, *p* = 0.038) and social trust (*β* = 0.021, *p* = 0.049) were not statistically significant after controlling the false‐discovery rate. Similarly, the hypothesized positive indirect path via social status (*β* = 0.018, *p* = 0.077) was not statistically significant.

Turning to gender's indirect effects, we first examined those mediated via height (Gender → Height → Mediator → Life Satisfaction pathway). Supporting our hypotheses, significant negative indirect effects were confirmed after FDR correction for the pathways involving safety (*β* = −0.041, *p* = 0.003) and financial satisfaction (*β* = −0.031, *p* = 0.013). Furthermore, consistent with the hypotheses of no effect for these specific pathways, the indirect effects via social support (*β* = −0.017, *p* = 0.039) and social trust (*β* = −0.015, *p* = 0.050) were confirmed as non‐significant after correction. However, the hypothesized negative indirect effect via social status (*β* = −0.013, *p* = 0.077) was not statistically significant, thus not supporting this specific hypothesis.

Next, we examined gender's direct psychosocial mediation pathways (Gender → Mediator → Life Satisfaction pathway). The hypothesized positive indirect effect via social support (*β* = 0.037, *p* = 0.011) was confirmed after FDR correction. In contrast to the hypothesis of no effect, a significant positive indirect effect was unexpectedly found via social trust (*β* = 0.027, *p* = 0.016) after correction. The hypothesized negative indirect effects via social status (*β* = −0.013, *p* = 0.128), financial satisfaction (*β* = −0.006, *p* = 0.703), and safety (*β* = 0.004, *p* = 0.790) were not statistically significant.

In sum, considering the 15 specific indirect pathways hypothesized in Table 1, seven hypotheses were supported by results significant after a FDR correction, while eight were not.

## 5 General Discussion

The present study aimed to investigate the complex relationships between height, gender, and life satisfaction, examining the potential mediating roles of five psychosocial factors: perceptions of safety, social trust, social support, social status, and financial satisfaction. Previous research has established a link between height and life satisfaction (e.g., Rees et al. 2009; Deaton and Arora 2009). Our study elucidates the nature of this relationship, with our multivariate SEM indicating that height's positive association with life satisfaction operates entirely through indirect psychosocial paths, as no residual direct effect remained once mediators were included. While the direct bivariate correlation between height and life satisfaction was modest (*r* = 0.11 in the pooled sample; 0.14 for men, 0.13 for women), its magnitude is comparable to other objective factors considered important for life satisfaction, such as marital status or social interaction frequency (Lucas and Dyrenforth 2006). Gender, by contrast, showed a nonsignificant zero‐order relation with life satisfaction, yet displayed significant links to psychosocial mediators, with both advantageous effects and disadvantageous effects that may counterbalance each other.

A key finding from our multivariate SEM analysis was that height demonstrated significant positive associations with all five psychosocial factors examined. Even the associations with social support and social trust, which were negligible in the zero‐order correlation matrix, became significant once gender and the other covariates were held constant. This pattern may reflect the differing theoretical pathways outlined earlier, where height's links to support and trust were expected to be more indirect. Crucially, the combined indirect effect of the five mediators fully explained the relationship between height and life satisfaction. After FDR correction, perceived safety (*β*_indirect = 0.059) and financial satisfaction (*β*_indirect = 0.044) emerged as the most robust channels, whereas the anticipated status route fell short of corrected significance. One plausible reason is that our status scale tapped self‐evaluated achievements (“more successful than others”) rather than the respect/admiration facet emphasized in sociometric‐status theories (Anderson et al. 2015; Fors Connolly and Johansson Sevä 2018).

Turning to gender, the SEM revealed a more complex pattern compared to height. While men reported higher social status, women reported significantly higher levels of social support and, unexpectedly, higher social trust (*β* = 0.153, *p* = 0.001) based on the direct path analysis. This mixed profile of psychosocial associations may help explain the often‐observed “gender‐life satisfaction paradox” (Batz and Tay 2018): why men and women frequently report similar life satisfaction levels despite documented female disadvantages in socioeconomic domains. Our mediation analysis confirmed that women experience higher life satisfaction indirectly via higher perceived social support and, unexpectedly, via higher social trust, with both pathways remaining significant after FDR correction. The finding regarding women's higher social trust contradicts our initial hypothesis of no effect, which suggested no relationship between gender and trust. However, this effect appeared specifically within our multivariate SEM framework where height is controlled. Prior research often examines the gender‐trust relationship without controlling for height. Therefore, the positive association observed here for both the direct Gender → Trust path and the indirect Gende → Trust → life satisfaction path might represent an underlying difference that becomes statistically apparent only when women's lower average height is accounted for. Furthermore, although not statistically significant, the small positive direct path from being female to life satisfaction in the final model hints that other factors, such as potentially different social comparison processes (Batz and Tay 2018), might also play a role, suggesting women might report slightly higher life satisfaction than men given the same psychosocial conditions.

The multivariate analysis also illuminated the interplay between height and gender. Specifically, the SEM indicated that height plays a role in explaining some apparent gender differences in psychosocial factors observed bivariately. The disappearance of direct gender effects on perceived safety and financial satisfaction once height was included in the model suggests that height differences between men and women may account for these specific disparities. The significant indirect paths found, where gender connects to life satisfaction via height's effect on safety and financial satisfaction, support this interpretation. This indicates that women's lower average height, potentially reflecting greater physical vulnerability (Manson et al. 2023) and fewer height‐related socioeconomic advantages, rather than gender per se, might underpin the lower perceived safety and financial satisfaction observed before controlling for height.

While our findings align with the interpretation that height confers social and psychological advantages leading to better psychosocial outcomes, it is important to consider alternative or complementary explanations for the observed height–psychosocial associations. For instance, underlying genetic factors could potentially influence both physical stature and certain personality traits or health predispositions relevant to psychosocial functioning. Furthermore, early life conditions, such as nutrition and health, which may be crucial determinants of adult height, may also exert independent, long‐lasting effects on life satisfaction. Our research design cannot in itself disentangle these pathways from the direct social effects of height. Future research using longitudinal data or genetically informed designs could help clarify the relative contributions of these different mechanisms. Nevertheless, the consistent patterns observed here underscore the relevance of physical stature as a correlate of important psychosocial experiences and life satisfaction.

Beyond the focal predictors, our analysis including immigrant status and age as confounders provided additional context. Immigrant status was negatively associated with social trust, perceived safety, social support, and financial satisfaction, aligning with previous research on the challenges faced by immigrants (Bayard‐Burfield 1999) and suggesting potential social and economic integration difficulties in Sweden. Paradoxically, immigrants reported higher perceived social status than native‐born Swedes, possibly reflecting different reference group comparisons. Age displayed complex relationships, correlating positively with social trust and financial satisfaction but negatively with social support, while showing no significant link to perceived safety or social status in our model. These age‐related patterns may reflect cumulative life experiences and developmental processes. The positive correlation between age and social trust aligns with previous studies (Li and Fung 2013) might stem from older adults' accumulated social competence and improved ability to discern trustworthiness in others. Similarly, higher financial satisfaction among older individuals could result from wealth accumulation over time and potentially adjusted financial expectations. The negative relationship between age and perceived social support may be attributed to age‐related changes in social networks, including retirement, loss of peers, and reduced family proximity. The absence of significant associations between age and both perceived safety and social status suggests these perceptions may be more strongly influenced by individual differences (e.g., personality traits) rather than age‐related developmental processes.

Although we did not specifically hypothesize the relative strength with which each psychosocial factor would relate to life satisfaction, a somewhat unexpected finding in our analysis was the relatively lower importance of social relations (social support and trust) compared to financial satisfaction and safety perceptions in predicting life satisfaction. This pattern appears to contrast with substantial literature positioning social relationships as primary drivers of subjective well‐being (e.g., Diener and Seligman 2002). This apparent discrepancy may be explained by our focus on life satisfaction rather than emotional well‐being. Cognitive evaluations of life tend to be more strongly related to perceptions of material conditions (and perhaps also safety perceptions), while emotional well‐being may show stronger connections to social experiences (Fors Connolly and Gärling 2024). Our findings suggest that while social factors remain important correlates to life satisfaction, in our Swedish sample, their importance appears secondary to perceptions of financial security and physical safety. This pattern partially aligns with need frameworks that position security needs as foundational (Maslow 1943).

Our findings contribute to the literature on subjective well‐being determinants by offering a more nuanced understanding of how height and gender relate to life satisfaction via distinct psychosocial pathways. By analyzing height and gender simultaneously, we provide a clearer picture than studies examining them in isolation, potentially reconciling conflicting findings. The results challenge the predominant focus on gender, ethnicity, and class in social inequality research, suggesting that other sources of inequality, such as height, may be overlooked yet significantly related to psychosocial outcomes and well‐being. Our study underscores the need to broaden social psychological inequality research and points to physical height as a variable warranting attention, as it correlated more consistently with psychosocial factors and life satisfaction than gender in our sample.

For advancing a mechanistic understanding of inequality, a step forward is distinguishing between effects directly linked to established individual characteristics (e.g., gender) and those stemming from correlated characteristics, whether socioeconomic, behavioral, or physical like stature. The theoretical model we propose extends beyond height and gender, offering a framework for investigating how other stable personal characteristics, such as body weight, physical attractiveness, or disabilities, may influence life satisfaction through similar psychosocial mechanisms. This approach could facilitate a more integrated understanding of how physical and social attributes jointly shape well‐being across diverse populations.

### 5.1 Limitations and Strengths

The cross‐sectional design limits causal inference, primarily by preventing establishment of temporal sequence. This raises potential reverse causality concerns between psychosocial factors and life satisfaction. However, since height and gender are stable characteristics typically established before the measured mediators and the outcome, reverse causality is less plausible for these specific predictors. This temporal stability of the independent variables makes the mediation analysis somewhat more justified, despite the cross‐sectional limitations.

Our reliance on self‐report measures introduces potential biases, though self‐reports are arguably appropriate for subjective constructs and demonstrated acceptable validity in our analyses (discriminant validity, Harman's test). We also acknowledge that while self‐reported height is generally valid for population studies (Gorber et al. 2007; Spencer et al. 2002), some reporting bias exists. We also acknowledge that we did not focus on gender‐height interactions; while our exploratory split‐file analysis suggested some moderation of the height‐life satisfaction link, formal moderation testing in our complex model was statistically inadvisable due to power limitations with relatively modest correlations (Chaplin 1991; Fairchild and MacKinnon 2009). Despite these limitations, the study has strengths, including a large, representative sample with a reasonable response rate, the use of reliable multi‐item scales for a broad range of latent factors, and validity testing across genders.

### 5.2 Practical Implications

Beyond theory, our findings may offer some practical insights. They highlight that stable characteristics like height are consistently linked to crucial psychosocial factors affecting life satisfaction, suggesting societal efforts addressing well‐being disparities should consider a wider array of attributes beyond traditional social categories. Overlooking factors like height may yield an incomplete picture of psychosocial inequality. Our findings suggest interventions could target safety perceptions, trust, support, status, and financial satisfaction to improve life satisfaction, potentially buffering disadvantages associated with shorter stature. The finding that height differences may explain some gender disparities in safety and financial satisfaction suggests interventions in these areas should consider factors beyond gender alone, such as perceived vulnerability or height‐related biases. However, these suggestions remain tentative and require further research and replication to confirm their validity and effectiveness; strong practical conclusions should not be drawn prematurely, particularly given the need to test the robustness of these specific pathways.

## 6 Conclusion

In conclusion, this study provides evidence for the mediating roles of psychosocial factors in the relationships between height, gender, and life satisfaction. Height demonstrated mostly positive associations with these factors, which fully accounted for its indirect positive association with life satisfaction. Gender's effects were more mixed, involving both psychosocial advantages and disadvantages that potentially counterbalance each other, leading to no net overall effect on life satisfaction. The analysis highlights the importance of considering physical characteristics like height alongside gender in well‐being research and underscores the complex interplay of factors shaping individuals' psychosocial experiences and overall life satisfaction.

## Author Contributions

The sole author was responsible for all aspects of this research, including the conceptualization, methodology, data analysis, and writing of the manuscript.

## Ethics Statement

Ethical approval was not required for the study because data was collected using a questionnaire without questions classified as sensitive by the Swedish Act concerning the Ethical Review of Research Involving Humans (2003:460). The purpose of the study was clearly described to participants and informed consent was obtained. Participants were completely anonymous, and the study did not seek to influence them. Studies fulfilling these criteria are exempt from ethics review according to regulations in Sweden.

## Consent

All participants received written information about the study, including its purpose, voluntary nature, and data handling procedures. In line with ethical guidelines for anonymous survey research without questions classified as sensitive, explicit written consent was not collected.

## Conflicts of Interest

The author declares no conflicts of interest.

## Data Availability

Data are available on request from the authors.

## Appendix A

**TABLE A1:** Demographic characteristics of the study sample (*N* = 990).

Characteristic	Category	*N*	%
Gender	Female	523	52.8
	Male	467	47.2
Born in Sweden	No	161	16.3
	Yes	829	83.7
Marital status	Married/Civil partnership	437	44.1
	Never married/in civil partnership	339	34.2
	Separated/Divorced/Widowed	207	20.9
	Missing/NA	7	0.7
Income group^a^	Lowest (Deciles 1–3)	141	14.2
	Middle (Deciles 4–7)	426	43
	Highest (Deciles 8–10)	400	40.4
	Missing/NA	23	2.3
Age group (years)	15–34	237	23.9
	35–54	263	26.6
	55–74	342	34.5
	75+	148	14.9
Total		990	100.0^b^

^a^ Income groups based on self‐reported household total net income deciles.

^b^ Percentages may not sum perfectly to 100.0 due to rounding.

**TABLE A2:** Standardized factor loadings for latent variables.

Latent variable	Indicator	Standardized factor loading
Life satisfaction	Life satisfaction 1	0.843
	Life satisfaction 2	0.772
Social trust	Trust 1	0.772
	Trust 2	0.886
	Trust 3	0.713
Safety	Safety 1	0.479
	Safety 2	0.948
Social support	Social support 1	0.763
	Social support 2	0.739
	Social support 3	0.559
Financial satisfaction	Financial satisfaction 1	0.851
	Financial satisfaction 2	0.549
	Financial satisfaction 3	0.630
Social status	Social status 1	0.788
	Social status 2	0.774

*Note:* Wordings for the items are displayed in Table 2.

**TABLE A3:** Fornell‐Larcker criterion for discriminant validity.

Construct	Life satisfaction	Social trust	Safety	Social support	Financial satisfaction	Social status
Life satisfaction	**0.808**					
Social trust	0.462	**0.794**				
Safety	0.569	0.385	**0.751**			
Social support	0.46	0.338	0.408	**0.693**		
Financial satisfaction	0.601	0.268	0.433	0.35	**0.689**	
Social status	0.35	−0.003	0.213	0.19	0.485	**0.781**

*Note:* Diagonal elements (in bold) represent the square root of the Average Variance Extracted (√AVE). Off‐diagonal elements represent the correlations between the latent constructs. Discriminant validity is established as the diagonal element for each construct is larger than the off‐diagonal elements in the corresponding row and column.

**TABLE A4:** Assessment of measurement invariance across gender for the latent factors.

Model	*χ* ^2^ (df)	CFI	RMSEA	ΔCFI	ΔRMSEA	Δ*χ* ^2^ (Δdf)	*p*
Configural	328.99 (150)	0.963	0.049	—	—	—	—
Metric	343.09 (159)	0.962	0.048	0.001	−0.001	14.099 (9)	0.012
Scalar	385.78 (168)	0.955	0.051	0.007	0.003	42.682 (9)	< 0.001
Strict	414.72 (183)	0.953	0.051	0.002	0	28.949 (15)	0.016

Abbreviations: Δ, change in respective fit index compared to the previous model; *χ* ^2^, chi‐square; CFI, comparative fit index; df, degrees of freedom; RMSEA, root mean square error of approximation.

**TABLE A5a:** Correlations between height, psychosocial factors and life satisfaction among females.

Variable	1	2	3	4	5	6	7	8
1. Life satisfaction								
2. Social trust	0.53***							
3. Safety	0.63***	0.46***						
4. Social support	0.50***	0.39***	0.42***					
5. Financial satisfaction	0.60***	0.30***	0.47***	0.37***				
6. Social status	0.39***	0.01	0.22***	0.22***	0.41***			
7. Height	0.13**	0.15***	0.19***	0.19***	0.04	0.03		
8. Immigrant	−0.10*	−0.15***	−0.15***	−0.20***	−0.10*	0.13**	−0.16***	
9. Age	0.16***	0.23***	0.07	−0.14**	0.03	−0.04	−0.13**	−0.09*

*Note:* **p* < 0.05, ***p* < 0.01, ****p* < 0.001.

**TABLE A5b:** Correlations between height, psychosocial factors and life satisfaction among males.

Variable	1	2	3	4	5	6	7	8
1. Life satisfaction								
2. Social trust	0.37***							
3. Safety	0.51***	0.34***						
4. Social support	0.43***	0.26***	0.48***					
5. Financial satisfaction	0.62***	0.26***	0.40***	0.38***				
6. Social status	0.33***	−0.00	0.17**	0.22***	0.53***			
7. Height	0.14**	0.02	0.12*	0.11*	0.19***	0.14**		
8. Immigrant	−0.16**	−0.05	−0.11*	−0.16**	−0.11*	0.10	−0.20***	
9. Age	0.22***	0.06	−0.04	−0.21***	0.17**	0.02	−0.01	−0.08

*Note:* **p* < 0.05, ***p* < 0.01, ****p* < 0.001.

**TABLE A6:** Effects of gender and height on psychosocial factors and life satisfaction.

Dependent variable	Predictor	Std. err	*z*‐value	*P*(>|*z*|)	*β*
Height	Gender (female)	0.422	−30.954	0.000	−0.700
Social trust	Gender (female)	0.179	3.220	0.001	0.153
	Height	0.010	2.493	0.013	0.120
	Immigrant	0.175	−2.365	0.018	−0.081
	Age	0.003	4.396	0.000	0.150
Safety	Gender (female)	0.043	0.293	0.770	0.014
	Height	0.002	3.932	0.000	0.207
	Immigrant	0.043	−2.667	0.008	−0.097
	Age	0.001	0.875	0.382	0.031
Social support	Gender (female)	0.060	4.423	0.000	0.222
	Height	0.003	2.910	0.004	0.148
	Immigrant	0.059	−5.015	0.000	−0.183
	Age	0.001	−4.898	0.000	−0.176
Financial satisfaction	Gender (female)	0.079	−0.412	0.680	−0.021
	Height	0.004	2.875	0.004	0.145
	Immigrant	0.077	−2.178	0.029	−0.079
	Age	0.001	2.600	0.009	0.093
Social status	Gender (female)	0.065	−2.277	0.023	−0.114
	Height	0.004	3.078	0.002	0.157
	Immigrant	0.064	3.506	0.000	0.128
	Age	0.001	0.111	0.912	0.004
Life satisfaction	Gender (female)	0.126	1.612	0.107	0.065
	Height	0.007	0.377	0.706	0.015
	Social trust	0.031	4.663	0.000	0.175
	Safety	0.172	6.008	0.000	0.286
	Social support	0.113	3.846	0.000	0.167
	Financial satisfaction	0.096	6.314	0.000	0.304
	Social status	0.102	2.813	0.005	0.118
	Immigrant	0.126	−0.256	0.798	−0.008
	Age	0.002	5.084	0.000	0.152
